# Exploring medical students’ barriers to reporting mistreatment during clerkships: a qualitative study

**DOI:** 10.1080/10872981.2018.1478170

**Published:** 2018-05-31

**Authors:** Melody P. Chung, Christine K. Thang, Michelle Vermillion, Joyce M. Fried, Sebastian Uijtdehaage

**Affiliations:** Deans Office, David Geffen School of Medicine at UCLA, Los Angeles, California, USA

**Keywords:** Medical student, mistreatment, reporting, barriers, qualitative, focus groups

## Abstract

**Background:** Despite widespread implementation of policies to address mistreatment, the proportion of medical students who experience mistreatment during clinical training is significantly higher than the proportion of students who report mistreatment. Understanding barriers to reporting mistreatment from students’ perspectives is needed before effective interventions can be implemented to improve the clinical learning environment.

**Objective:** We explored medical students’ reasons for not reporting perceived mistreatment or abuse experienced during clinical clerkships at the David Geffen School of Medicine at UCLA (DGSOM).

**Design:** This was a sequential two-phase qualitative study. In the first phase, we analyzed institutional survey responses to an open-ended questionnaire administered to the DGSOM graduating classes of 2013–2015 asking why students who experienced mistreatment did not seek help or report incidents. In the second phase, we conducted focus group interviews with third- and fourth-year medical students to explore their reasons for not reporting mistreatment. In total, 30 of 362 eligible students participated in five focus groups. On the whole, 63% of focus group participants felt they had experienced mistreatment, of which over half chose not to report to any member of the medical school administration. Transcripts were analyzed via inductive thematic analysis.

**Results:** The following major themes emerged: fear of reprisal even in the setting of anonymity; perception that medical culture includes mistreatment; difficulty reporting more subtle forms of mistreatment; incident is not important enough to report; reporting process damages the student–teacher relationship; reporting process is too troublesome; and empathy with the source of mistreatment. Differing perceptions arose as students debated whether or not reporting was beneficial to the clinical learning environment.

**Conclusions:** Multiple complex factors deeply rooted in the culture of medicine, along with negative connotations associated with reporting, prevent students from reporting incidents of mistreatment. Further research is needed to establish interventions that will help identify mistreatment and change the underlying culture.

## Background

Student mistreatment remains a widespread problem in medical schools in the USA and across the world. Mistreatment can have deleterious effects on medical students’ emotional well-being and professional attitudes [1,2]. Specifically, medical students who are subject to abusive behaviors are more likely to experience mental health issues such as post-traumatic stress [3,4], depression and low career satisfaction [5], and even suicidality [6]. More than three decades of studies have shown that the behavior of faculty, residents, and nurses toward medical students can be unprofessional and abusive, particularly during clinical clerkship rotations [1,5,7,8]. A meta-analysis demonstrated a high prevalence of harassment and discrimination toward medical trainees worldwide, with 59% of medical trainees experiencing at least one form of harassment or discrimination during their training [8].

The proportion of students who experience mistreatment is significantly higher than the proportion of students who report mistreatment to a designated faculty member or a member of the medical school administration empowered to handle such complaints. According to the Association of American Medical Colleges (AAMC) Graduation Questionnaire, 38% of US medical students have experienced some form of mistreatment or discrimination at least once in their medical student education [9]. Among those who said they were mistreated, 80% of students stated they chose not to formally report the incident to someone at their institution.

These national statistics reflect survey results at our own institution [10]. Reluctance to report mistreatment may very well contribute to a persisting culture of abuse despite our multi-pronged efforts to eradicate it [10]. Our efforts include publicizing clear institutional policies on harassment and discrimination, and developing multiple safe mechanisms to anonymously report mistreatment. Specifically, our medical students are informed of the following pathways to report both directly experienced or secondarily witnessed mistreatment: confidential course evaluations for each clerkship; anonymous feedback surveys administered by the Medical Student Council; a Well-Being Survey administered at the end of the third year; direct contact with the clerkship coordinator or medical school administration (including the Gender and Power Abuse Committee; and/or direct contact with the UCLA Office of Ombuds Services, Title IX office, or UCLA Office of Equity, Diversity, and Inclusion).

Mistreatment that is not reported or documented is effectively non-extant, as it cannot be acted upon and thus may perpetuate an unsafe clinical learning environment. To our knowledge, no research exists to date that studies medical students’ reluctance to report mistreatment whether it was experienced first-hand or secondarily witnessed. Examining why students choose not to report mistreatment is paramount to effectively respond to unprofessional behavior and develop policies to eradicate mistreatment. In this qualitative study we explored third- and fourth-year medical students’ reasons for not reporting mistreatment during clinical clerkships using survey data and focus groups.

## Methods

### Study team

Two of us (MC and CT) initiated this research effort as senior medical students. At the time of study, MC and CT served on the Medical Student Council, an organization that aims to positively influence student life and facilitate communication between the student body and the school leadership. Both had secondarily witnessed mistreatment during clerkships. JF is assistant dean and chair of the Gender and Power Abuse Committee at DGSOM and thus plays a central role in monitoring student mistreatment. SU was director of research and oversaw the research-technical aspects of the study. MV is a senior administrative analyst with extensive qualitative research experience.

### Study design

The UCLA Institutional Review Board approved our protocol. In our study, we employed a sequential two-stage qualitative approach involving both a survey and focus groups. The focus group methodology was chosen to capture participants’ perspectives as they considered their views while interacting with others, which would not be portrayed by survey results alone.

#### Definitions of mistreatment

At our institution, we define mistreatment as follows in our policy handbook and provided these definitions to all students in this study: (1) physical mistreatment (defined as ‘slapped, struck, pushed’), (2) verbal mistreatment (defined as ‘yelled or shouted at, called a derogatory name, cursed, ridiculed’), (3) sexual harassment (defined as ‘inappropriate physical or verbal advances, intentional neglect, sexual jokes,’ and ‘mistreatment based on sexual orientation’), (4) ethnic mistreatment (defined as ‘intentional neglect, ethnic jokes, comments and expectations regarding stereotypical behavior’), and (5) power mistreatment (defined as ‘made to feel intimidated, dehumanized, or had a threat made about a recommendation, your grade, or your career’).

#### Phase one: analysis of institutional survey data

In the spring of 2015, we reviewed archived, anonymous DGSOM Well-Being Survey data (described elsewhere [10]) from the graduating classes of 2013–2015. We administer this survey annually to the rising senior students to monitor the extent of mistreatment in clerkships. In one portion of the survey, students are encouraged to provide an open response to five variations of the following question: ‘If you experienced [physical/verbal/sexual/ethnic/power] mistreatment, but didn’t seek help, why not?’ The overall Well-Being Survey response rate was 98.8% (477 out of 483 students) over three years, with 25.8% of students (123 out of 477) leaving at least one comment across the five questions listed above. The response rates between different graduating classes were comparable.

Our senior data analyst (MV) compiled the multi-year survey data into a single Microsoft Excel spreadsheet for analysis. MV, who routinely codes these data for internal reporting of the survey results, recused herself from initial coding so as to not bias the resulting themes. Two members (MC and CT) independently read the raw data several times to identify themes and categories using inductive content analysis [11]. After discussion, a coding frame was developed and the data were coded independently. If new codes emerged, the coding frame was changed and the transcripts were reread according to the new structure. This process was used to develop categories, which were then conceptualized and finalized into broader themes after further discussion. We then compared the themes that MC and CT found in the multi-year data set to those from MV’s independent internal reports from each year for triangulation. Any discrepancies were resolved through discussion until consensus was achieved. For each theme, we quantitatively tallied the frequencies in which students commented. This provided us with a preliminary understanding of the reasons why students fail to report mistreatment. Based on these findings, we formulated focus group probing questions listed in Table 1.

**Table 1.** Focus group probing questions.

1. What barriers to reporting mistreatment do you think medical students may have?
2. Under what circumstances would you choose to report mistreatment?
3. Do you feel like you have a clear understanding of what mistreatment is or isn’t?
4. Would anonymous reporting mechanisms help overcome the fear of reprisal?
5. Some students have not reported in the past because mistreatment seems part of the culture of medical education. What is your opinion about that?

#### Phase two: focus groups

We conducted five 30–60 min focus group discussions between February and April 2015 to further explore students’ experiences with mistreatment and their perspectives on reporting. Eligible participants were third- or fourth-year students (classes of 2015 and 2016) enrolled in any of our MD and combined degree programs. MC sent all eligible participants (*N* = 362) an email invitation through electronic class distribution lists. We recruited additional participants through snowball sampling. Students were provided free lunch as an incentive for participation. Participation was voluntary and confidential, and verbal consent was obtained from all participants. Focus groups were led by MC, a senior medical student at the time of study. We hoped that a peer-to-peer exchange without the presence of an administrator or faculty member would uncover issues that would have otherwise remained hidden.

We started each focus group by providing definitions of each form of mistreatment as listed previously. We opened the discussions with a general, open-ended question: ‘Many medical students experience mistreatment but choose not to report it. What barriers to reporting mistreatment do you think medical students have?’ Interview probes developed from survey responses (Table 1) were used to stimulate in-depth discussions. After each focus group, MC followed up with a post-focus group questionnaire via email, collecting demographic information and asking privately in a ‘yes or no’ format if students had directly experienced mistreatment, and if so, whether or not they had reported it. This post-focus group questionnaire was conducted to gather these data in case there were situations where students felt uncomfortable sharing their personal experiences with mistreatment in front of their peers.

#### Data analysis

We audiotaped discussions and created a de-identified verbatim transcript after each focus group to analyze data on an ongoing basis. Two members (MC and CT) of the research team independently read and coded the interviews using first-level provisional coding. Through iterative readings of the transcripts, similar codes were grouped to reduce the number of categories and major concepts were identified [12,13]. The major concepts were further defined, developed, and refined into main themes. All parent transcripts were analyzed and coded iteratively after each focus group. Focus groups were stopped after five groups, as saturation of major themes occurred with no new significant material occurring during the fourth and fifth focus groups. All themes were sent to one randomly selected participant from each group for member checking to confirm that they accurately reflected their perspectives, with participants providing input on phrasing.

## Results

### Well-being survey results

Themes resulting from the survey analysis are listed in Table 2. The most frequently cited themes for not reporting mistreatment included: incident did not seem important enough to report, fear of reprisal, reporting process requires too much time and effort, mistreatment seems to be a part of medical education and culture, issue was resolved by the student, and being unsure if the incident was considered mistreatment. Although most survey comments were short and brief, these survey results provided a foundational basis to create focus group probing questions.

**Table 2.** Themes regarding non-reporting of mistreatment emerging from well-being survey from classes of 2013–2015.

Reasons for not reporting	Total number of responses	Percentage of all responses
The incident did not seem important enough to report	54	28
Fear of reprisal	43	22
The reporting process requires too much time and effort	20	10
Mistreatment seems to be a part of medical education culture	14	7
I resolved the issue myself	10	5
I was unsure if the incident was considered mistreatment	6	3
It seemed like the person did not intentionally mean to be harmful or was joking	5	3
I did not know what to do	4	2
The incident did not affect me directly	4	2
No one would believe my report	3	2
I could not identify the person’s name	2	1
Total	197	100

### Focus group findings

We conducted five focus groups, each with an average of six participants per group (range: 4–10 participants) with a total of 30 of 362 eligible participants. A total of 19 of the 30 students (63%) reported having felt mistreated. Only 9 (30%) participants who had felt mistreated officially reported an incident to the administration. Further demographics of focus group participants are included in Table 3. Focus group discussions included several personal narratives, and students who did not directly experience mistreatment were still able to provide insight on secondarily witnessed mistreatment. We grouped the themes as follows: cultural barriers related to the inherent hierarchy of medicine, extrinsic barriers related to the source of mistreatment, and intrinsic barriers related to the student. In the following sections, we have highlighted students’ quotes from the focus groups to illustrate major themes. We have lightly edited the quotes so that they are more readable, but we have not changed their style or meaning. Medical student quotations are denoted as 'MS3' (third-year medical student) or 'MS4' (fourth-year medical student) depending on the source.

**Table 3.** Demographics of focus group participants (n = 30).

	Number of Participants (percentage of n)	
Medical School Year		
Third Year	12 (40)	
Fourth Year	18 (60)	
Gender		
Male	13 (43)	
Female	17 (57)	
Did you personally experience mistreatment on clerkships?		
Yes	19 (63)	
No	11 (37)	
If you experienced mistreatment, what types of mistreatment did you experience?		
Verbal	9	
Power	8	
Physical	6	
Ethnic	2	
Sexual	1	
If you experienced mistreatment, did you report it through any mechanism?		
Yes	9	
No	10	
If you experienced mistreatment, who mistreated you?		
Residents	5	
Attendings	13	
Nurses	2	

## Cultural barriers related to the inherent hierarchy of medicine

### Fear of reprisal

A common theme that emerged from all focus groups was the fear of reprisal. Students were hesitant to report mistreatment because of the concern that faculty, residents, and nurses could negatively impact their evaluations and their future careers. Students were particularly fearful of reporting mistreatment in their intended specialties, lest they damage future relationships and connections. Surprisingly, most students were unlikely to report even if completely anonymous reporting mechanisms were employed. Some students were unlikely to report even after a delay in timing after clerkship completion, or in some cases, even after graduating from medical school. Many students agreed that the details and context of a particular incident would be sufficient evidence to identify the reporting student, even if no names were used, since only a limited number of students rotate in a particular specialty at a clinical site.

Fear of retribution is probably the greatest barrier on everyone’s mind. Obviously if you’re reporting it within the year that you’re doing your clerkship, it can be easily traced back to you because the incident is so fresh. The community is so small. Anyone who speaks up is labeled as a whistleblower. – MS4

### Perception that mistreatment is part of medical culture

Many students expressed that they did not report mistreatment because they had become acculturated to have the expectation that mistreatment is part of medical education and culture. Students ‘learned their place’ in occupying the lowest tier of the medical hierarchy, citing the power differential as a major reason as to why they were especially vulnerable to mistreatment by residents and attending physicians. One student called medical school a ‘hazing process,’ and had come to accept mistreatment because ‘that’s just the way it is.’ To cope with this learned powerlessness, many students adopted the belief that they needed to develop resilience in order to excel on clinical clerkships. Multiple students in one focus group nodded in agreement upon hearing the following comment from a student:

When it comes to me, I didn’t report most things because I’m like, ‘I need to man up.’ – MS4

Furthermore, some students believed that certain faculty members perpetuated a culture of mistreatment by refusing to take reports of mistreatment seriously. In one focus group, multiple students were upset when a course director brushed off complaints about a particular individual during an open course feedback session, especially when there were genuine concerns about widespread mistreatment. The lack of response to reports of mistreatment undermined students’ confidence in the leadership’s ability and commitment to effect change. This discouraged many students from reporting further episodes of mistreatment on the clerkship, as some students felt that ‘nothing would be done about it.’ As one student quipped:

At a feedback session, a student said one certain attending was mistreating all the medical students there. The course director said: ‘That’s just their personality, you just have to get used to that.’ No, that’s not right. – MS3

### Difficulty reporting more subtle forms of mistreatment

Despite our institution’s efforts to define mistreatment, many students reported feeling confused about gray areas that do not cleanly fall under the formal definitions. Students felt confident in their ability to report ‘blatant mistreatment,’ such as physical mistreatment or sexual harassment, since these were considered tangible acts of abuse that warranted reporting. In contrast, students were less inclined to report incidents that appeared to be more equivocal, such as power, verbal, ethnic mistreatment, or neglect. Many students across different focus groups mentioned that there are varying individual thresholds for what they consider mistreatment, as individuals may interpret the same incident differently. As one student quipped:

It’s really obvious if someone slaps you in the face or sexually harasses you, or like stabs you with a scalpel, that’s pretty obvious mistreatment. I feel like I know what blatant mistreatment is, but then things like ignoring you, not talking to you, not teaching you, or saying things to you that make you feel bad about yourself – those all happen to you in real life – am I supposed to report that? – MS4

## Extrinsic barriers related to source of mistreatment

### Concern about damaging the student–teacher relationship

Students were not only concerned about harming their own careers through effects of retaliation, but they also worried that reporting mistreatment would cause deleterious career repercussions for individuals being reported. Many students hoped to improve the clinical learning environment by offering suggestions on how to make teachers more effective, but not at the expense of harming their careers. Some students also worried that the reporting process would negatively affect the student–teacher relationship by causing unnecessary levels of restraint for teachers interacting with students, or worse yet, inadvertently lessen the rigor of education for students:

A nice attending told me she’s afraid of grilling medical students out of fear of being reported. I wish they would ask me questions, I love it when attendings talk to me. She was keeping me at arm’s length out of fear of being too tough on me. – MS4

Another student had problems with the entire model of anonymous reporting because it prevented students from having open communication with their mentors.

I can only imagine how much attendings hate it when medical students don’t open honest dialogue with them and just destroy them in the final evaluation. The worst part of reporting is that nobody wins. – MS4

### Empathy with the source of mistreatment

Some students did not report mistreatment because they presumed underlying reasons for abusive behaviors, such as workplace-related or personal-related stress, and empathized with these individuals in these situations. A couple of students hypothesized that certain specialties with a higher prevalence of mistreatment may have multiple factors contributing to increased stress or burnout levels, such as higher litigation rates or more hours worked. One particular student felt strongly that students had no right to judge physicians for their actions, but instead should try to understand the context surrounding their stress:

We seem to imagine ourselves as pinnacles of equality, compassion, and moral justice. We forget that physicians are, at the end of the day, just people. Just like your next-door neighbor, people in the medical profession are just as privy to fatigue, worries, and stress. And this stress manifests in many different ways. – MS4

### Incident was deemed not important enough to report

Some students did not report mistreatment because some incidents were perceived to be minor, unintentionally harmful, or done in a joking manner. Students acknowledged that the reporting of any abuse or mistreatment is to some extent subjective and depends on the personality and psychosocial context of the student. Most students, however, felt that they had relatively high thresholds for calling an incident ‘mistreatment’ and thus did not feel the need to report any minor incidents. As one student stated:

I claimed that I had never experienced mistreatment before. That’s after having been called North Korean, being confused with other Asians, being told I look like a Japanese baby. So why do I not count myself as mistreated? Because I just don’t care. It’s only mistreatment if you let yourself be affected. – MS4

## Intrinsic barriers related to student

### Reporting process requires too much time and effort

Many students shared that they were too exhausted from the demands of rotating through clerkships and studying for exams to justify the time and effort to report a single event that may only affect them transiently, as they would quickly rotate off service to a different rotation.

For a lot of the mistreatment it’s small enough where reporting it is too much work. I have to describe it in detail, I’d rather just deal with it. I’m tired, I’d rather go sleep or study for my shelf exam. – MS3

One student who did choose to report mistreatment to clerkship directors later expressed regret about reporting, stating that the process was too cumbersome and time-consuming as multiple in-person meetings with clerkship directors were required, inadvertently pulling the student away from clinical duties.

## Differing perspectives

We were surprised to learn that there were differing perspectives even among two consecutive generations of classes. Many students (mostly fourth-year medical students) viewed the reporting process negatively and were afraid that the reporting process would weaken the overall reputation of medical students at the institution:

The downside is that everyone thinks we’re too coddled. They joke about what they can or can’t do to medical students. They’re aware of the issue but in a bad way. They think we’re overly sensitive. – MS4

In contrast, there were students (mostly from the third-year medical class) who disagreed with the aforementioned statements, as they felt that reporting had overall improved the culture of the school for the vast majority of people:

When I was on one of my rotations, residents and attendings told me very clearly that they didn’t want to violate duty hours or mistreat us. It’s a positive reflection on the entire system because avoiding mistreatment is on people’s minds. – MS3

## Discussion

Our study takes a novel approach to understanding students’ reasons behind underreporting of mistreatment during clinical training, and, to our knowledge, it is the first study of its kind. In our two-stage approach, we leveraged the advantages of both a survey and focus groups, thus providing insights that could have been missed if either method was employed alone. In this exploratory qualitative study, we discovered that the reasons for not reporting mistreatment are multifaceted and deeply rooted in the culture and hierarchy of medicine, and that the solution to eradicating mistreatment may not be as simple as encouraging more reporting of events.

Even though our medical students across the board agreed that mistreatment had negatively affected their educational experiences, many of our students had become ‘acculturated’ to mistreatment and had learned to accept it as a part of their medical education. Thus, the perception that mistreatment is part of the fabric of medical education continues to undermine efforts to eradicate abuse. Prior studies have shown that unintended messages in the ‘hidden curriculum’ promulgated by unprofessional behavior in the learning and care environment negatively impact learners’ professional development, well-being, and empathy [6,14]. Because the problem is cultural and institutionalized, leaving a ‘transgenerational legacy’ [15], it can be particularly difficult to eradicate.

Furthermore, inherent in the culture of medicine is the hierarchy and power differential between trainees and trainers [16], with the latter group having control over the former group’s evaluations and grades. Fear of reprisal was one of the most commonly cited barriers to reporting mistreatment, and students’ fears were not assuaged by the promise of anonymous reporting mechanisms. To our surprise, this fear of reprisal transcended both space and time, as many participants stated that they would not report even in situations such as delayed reporting or even after graduating from medical school. Furthermore, except for a small minority of students, students were generally fearful about providing feedback directly to sources of mistreatment due to the inherent hierarchy of medicine, and instead, wanted to turn to faculty members or clerkship chairs who were available, understanding, and importantly, responsive. Many students were disappointed in faculty members who had witnessed or heard about mistreatment committed by their colleagues but did not intervene, as this tacitly condoned abusive behaviors and perpetuated the existing culture that mistreatment is acceptable and perhaps even expected [17].

Perhaps one approach to mitigate the inherent power differential between clinical teachers and students is to create a ‘firewall’ between clinical teachers who work directly with students on the wards and other independent faculty who make high-stake grading decisions. This could be accomplished in ‘programmatic assessment’ involving frequent low-stake assessments by clinical coaches that optimize learning, as advocated by van der Vleuten *et al*.[18]. The separation between clinical coaches and high-stake decisions may mitigate the fear of reprisal when students consider reporting mistreatment. Future research could compare reporting rates in such an environment to the more traditional approach in which clinical teachers partake in promotion decisions.

Consistent with the concept map described by Gan *et al*. at McGill University [19], our students perceived ‘mistreatment’ as a wide spectrum, with incident-based mistreatment (e.g., a verbal insult, physical abuse, or unwanted sexual conduct) on one end and environment-based mistreatment (e.g., mistreatment based on a suboptimal learning environment or attitude, such as a subjective feeling of being disrespected within a certain learning environment) on the other end. Incident-based mistreatment is considered blatant, single incident, easily reportable, and more empowering for students because of faculty support [19]. On the opposite end, environment-based mistreatment is more subjective, subtle, culturally embedded, difficult to report, dismissed by faculty, and can be more distressing for students [19]. Even though environment-based mistreatment may not fall cleanly under official policies or definitions of ‘official mistreatment’ when taken in isolation, repeated incidents in a learning environment may still lead to systematic mistreatment. Thus, it appears that underreporting of environment-based mistreatment may be occurring systematically at our institution because these types of incidents are more challenging for students and faculty to define and may fall into gray areas.

How does one change the culture of medicine to decrease student mistreatment? Because the problem is cultural and institutionalized, it is unlikely to disappear through any one intervention alone, and we believe that there are no easy answers. Rather, as a deeply ingrained cultural practice, mistreatment of medical students requires focused action to disrupt the existing culture and replace it with a ‘culture of compassion, tolerance, and respect’ [20]. Reforming the culture may require a top-down approach from leadership [20], rather than a bottom-up approach through reporting from students. Medical school leadership, including deans, department chairs, clerkship chairs, and residency training directors, must create a culture focused on patient safety, team-based care, and the well-being of the organizations’ members, while advocating a zero tolerance policy on bullying and mistreatment and responding to concerns from the student body [21]. Krugman *et al*. suggest that a ‘comprehensive, visible, high-priority organization commitment to culture change is necessary to promote candid communication up and down the academic hierarchy’ [20]. Until the culture of medicine affirms that broad input is vital, students are ‘unlikely to feel safe in expressing concerns, providing feedback, reporting mistreatment or unprofessional behaviors, and offering suggestions for improvement’ [20].

Furthermore, the problem of mistreatment could be reframed as a professionalism issue that highlights the responsibilities of administrators, teachers, resident, nurses, and also of students. Our findings clearly demonstrate that a student’s determination of whether behavior is unprofessional or not (and thus, the inclination to report it) is heavily influenced by the environmental and organizational context in which it occurs. All stakeholders, ranging from students, teachers, administrators, and national organizations, must make a concerted, joint effort to foster a psychologically safe learning environment for our medical learners in which mistreatment has no place. Lesser *et al*. conceptualize professionalism in terms of observable behavior both by individuals as well as the organization at large. Lesser *et al*. suggest concrete avenues to promote individual and organizational behaviors that align with core values of respect, integrity, pursuit of excellence, and fairness [22]. As we propose to extend their framework to the educational realm, it may mitigate the confusion and uncertainty that medical students expressed in our study regarding what constitutes ‘mistreatment.’

More controversially, students debated whether or not the process of reporting mistreatment was even beneficial to the learning environment. Most fourth-year medical students believed that the reporting process was overall harmful and inadvertently led to negative consequences in the learning environment, raising thought-provoking concerns that were not captured by responses in our Well-Being Survey or the AAMC Medical School Graduation Questionnaire [9]. These included concerns about damaging student–teacher relationships, lessening the rigor of education, preventing open dialogues with teachers, weakening the overall reputation of the medical school, and making students too sensitive or ‘not resilient enough.’ Most students from the third-year medical class disagreed, as they felt that increased reporting had already improved the culture of the school by bringing overall awareness on stopping the bullying culture of medicine. These diverging viewpoints may be explained by the fact that third-year medical students were more likely to have directly experienced changes from interventions to reduce mistreatment during core clinical clerkships.

Since the term ‘reporting’ may carry negative connotations for both students and faculty, promoting a culture of constant feedback through a mandatory bidirectional evaluation process may be a better alternative. As an intervention at our institution, students are now required to complete evaluations for all residents and attending physicians whom they have worked with on clerkship rotations. The evaluation forms now have the following ‘yes or no’ questions with an optional area for comments: ‘Did this individual treat you with respect?’ and ‘Did this individual treat others with respect?’ We hope that simple interventions like this will enable medical school faculty to identify individual outliers who may be committing unprofessional or abusive behavior, so that departments can help faculty members hone feedback and teaching skills that are more in line with the values at our institution. Meanwhile, students may learn, as they begin to form their professional identity, what constitutes professional behavior. Furthermore, faculty development may potentially help teachers create safe learning environments and develop approaches to teaching that do not rely on what is often perceived as humiliation or worse, as shaming [23]. Another intervention that we implemented post-study was the creation of resident teaching awards nominated by the medical student body. These awards recognize outstanding individuals at our institution who model exemplary behaviors toward medical students and provide an incentive for teaching. Further research is needed to determine if these interventions will help improve the clinical learning environment and decrease rates of mistreatment.

Our study is limited in that it is based on a single cohort of students at one institution collected over a short period of time, and student perspectives and institutional culture may have changed from the time of this study. Since focus group participants were voluntarily recruited, participants may have been more interested in or may have experienced more incidents of mistreatment compared to nonparticipants resulting in selection bias. Most of our participants (63%) reported experiencing mistreatment, but we could not independently verify these incidents. To avoid potential biases from having personally experienced mistreatment, the moderator did not share personal experiences during focus groups, member-checked themes with focus group participants, and triangulated results with survey responses. A major strength of this study is that the majority of our focus group participants included senior medical students who had already interviewed at or matched into residency programs, thus minimizing concerns for repercussions from participating in this study.

In conclusion, we discovered that multiple complex factors deeply rooted in the culture of medicine, along with negative connotations associated with reporting, prevent students from reporting incidents of mistreatment. Unless the culture of medicine changes to affirm that mistreatment is unacceptable, students are unlikely to feel safe in expressing concerns, providing feedback, and helping to identify mistreatment. Further research in this area is needed to develop interventions that will help uncover mistreatment, disrupt the existing bullying culture of medicine, and improve the clinical learning environment.
